# Preventive Interventions for Internet Addiction in Young Children: Systematic Review

**DOI:** 10.2196/56896

**Published:** 2024-08-30

**Authors:** Yansen Theopilus, Abdullah Al Mahmud, Hilary Davis, Johanna Renny Octavia

**Affiliations:** 1 Centre for Design Innovation Swinburne University of Technology Melbourne Australia; 2 Centre for Ergonomics Parahyangan Catholic University Bandung Indonesia; 3 Centre for Social Impact Swinburne University of Technology Melbourne Australia

**Keywords:** children, digital device, internet addiction, intervention, prevention, problematic internet use, technology, young children, problematic use, preventive, interventions, systematic review, internet, addiction, prevent, reduce, risk, risks, database, databases, child, PICOS, thematic analysis, Population, Intervention, Comparison, Outcome, and Study type

## Abstract

**Background:**

In this digital age, children typically start using the internet in early childhood. Studies highlighted that young children are vulnerable to internet addiction due to personal limitations and social influence (eg, family and school). Internet addiction can have long-term harmful effects on children’s health and well-being. The high risk of internet addiction for vulnerable populations like young children has raised questions about how best to prevent the problem.

**Objective:**

This review study aimed to investigate the existing interventions and explore future directions to prevent or reduce internet addiction risks in children younger than 12 years.

**Methods:**

The systematic review was conducted following the PRISMA (Preferred Reporting Items for Systematic Reviews and Meta-Analyses) guidelines. We searched for relevant literature from 4 research databases (Scopus, Web of Science, PubMed, and PsycINFO). We included 14 primary studies discussing the interventions to prevent or reduce internet addiction risks in young children and their efficacy outcomes.

**Results:**

The preventive interventions identified were categorized into four approaches as follows: (1) children’s education, (2) parenting strategy, (3) strategic physical activity, and (4) counseling. Ten interventions showed promising efficacy in preventing or reducing internet addiction risks with small-to-medium effect sizes. Interventions that enhance children’s competencies in having appropriate online behaviors and literacy were more likely to show better efficacy than interventions that force children to reduce screen time. Interventions that shift children’s focus from online activities to real-world activities also showed promising efficacy in reducing engagement with the internet, thereby preventing addictive behaviors. We also identified the limitations of each approach (eg, temporariness, accessibility, and implementation) as valuable considerations in developing future interventions.

**Conclusions:**

The findings suggest the need to develop more sustainable and accessible interventions to encourage healthy online behaviors through education, appropriate parenting strategies, and substitutive activities to prevent children’s overdependence on the internet. Developing digital tools and social support systems can be beneficial to improve the capability, efficiency, and accessibility of the interventions. Future interventions also need to consider their appropriateness within familial context or culture and provide adequate implementation training. Last, policy makers and experts can also contribute by making design guidelines to prevent digital product developers from making products that can encourage overuse in children.

## Introduction

### Internet Addiction in Young Children

The internet provides powerful functions and benefits in supporting human lives and work. Globally, people spend more than 6.5 hours daily on online activities, meaning we spend almost a third of our lives using the internet [1]. In this digital age, most children begin using the internet since early childhood (younger than 5 years) [2-5]. The existing guidelines suggest that parents do not give their children access to internet devices before they are 2 years old [6,7]. The increasing access to the internet for young children raises concern about the negative impacts of internet overuse that could lead to addictive behavior. This emerging phenomenon is often referred to as internet addiction [8,9]. Behavioral disorders related to the internet and gaming activities have been recognized as a diagnosable mental health condition that needs further studies in the *International Statistical Classification of Diseases* and the *Diagnostic and Statistical Manual of Mental Disorders* [10,11].

Internet addiction is identified as a behavioral disorder caused by the excessive and uncontrolled use of the internet and digital devices, which can lead to physical, mental, and social problems [12,13]. Internet addiction could bring many negative consequences for children, including mental health problems such as anxiety, emotional instability, and depression [14,15]; physical health problems such as headaches, eye problems, and musculoskeletal pains [16,17]; declining performance [18,19]; sleep disorder [20]; antisocial behavior [21,22]; speech delay [23]; and hindering child growth and development [24]. Prior studies highlighted that children are considered vulnerable to internet addiction [8,9,25]. In 2022, the estimated global prevalence of internet addiction in children was 13.82% [26]. Multiple studies highlighted some reasons that underlie the high risks of internet addiction in young children, such as limited self-control [18], incomplete brain development [20], parental limitations [27,28], and influence from children’s environment [29,30].

According to the Interactional Theory of Childhood Problematic Media Use, some distal, proximal, and maintaining factors jointly contribute to determining the risks of internet addiction in children younger than 12 years [31]. For context, the media referred to in the model is digital media that can be accessed and distributed through the internet [31]. The distal factors include the family socioeconomic conditions, family dysfunctions (eg, behavioral, academic, and social dysfunctions), and digital environments (eg, types of devices used, online activities, and content accessed). The proximal factors include the access, behavior, and attitude toward the internet and media use from the children, their family, and their peers. The maintaining factors include parent-child relationships, peers’ influence on the internet and media use, and self-efficacy and self-regulation in children. Internet addiction in young children becomes more complex than in adults since the people around them (eg, parents, siblings, or peers) may significantly influence their online behavior [31,32]. In addition, prior studies were concerned about product features that could encourage children to have more screen time, which can exacerbate the problem [33,34].

### Related Work and the Objective of This Study

This review study was conducted to fill gaps in the discussion of the current state and future directions of preventive interventions for internet addiction in young children. We identified the gaps from the prior studies. Vondráčková and Gabrhelík [25] and Lee et al [35] conducted review studies about the prevention of internet addiction. They discussed the topic from several aspects, such as conceptual model, target groups, specific skills, characteristics, and environmental (or social). However, they did not discuss the preventive interventions and their outcomes. They highlighted that research into and development of preventive interventions for internet addiction are still scarce, especially for vulnerable populations like children. The finding from Vondráčková and Gabrhelík [25] suggested the need for more intervention studies of children by involving their environment (eg, parents, teachers, and peers). Young children typically have unique characteristics related to their internet use and risky online behavior, thus requiring appropriate intervention developed for them [31,36]. The previous findings indicate the need to conduct more investigation on appropriate preventive interventions for internet addiction in children.

Prior studies reviewed the existing internet addiction treatments for various target groups. Xu et al [37] and Kuss and Lopez-Fernandez [9] discussed psychological treatments and therapies for internet addiction. Ayub et al [38] discussed treatment modalities for addressing internet addiction in children and adolescents. Those studies similarly investigated internet addiction treatments’ methods, domains, and effectiveness. The use of psychotherapies like cognitive behavioral therapy and electro-acupuncture were reported as promising treatments for reducing the symptoms of internet addiction [9,37,38]. However, there is a lack of studies discussing the preventive interventions for internet addiction in young children. Preventive interventions are essentially needed for children to prevent them from experiencing the negative consequences of internet addiction [8,25].

The high risks and prevalence of internet addiction in young children raised the urgency in exploring how best to prevent this problem. There is a need to understand how the existing preventive interventions have been developed, implemented, and assessed to prevent or reduce the risks of internet addiction in young children. Young children are categorized as persons 12 years and younger based on the theory of child cognitive development [39,40]. Children older than 12 years are generally classified as adolescents with different characteristics and internet behavior than younger children [31,40-42]. Therefore, this review study aimed to investigate the existing interventions and explore future directions to prevent or reduce internet addiction risks in children younger than 12 years. This review study contributed to filling the gaps in understanding the current approaches, efficacy outcomes, and strengths and limitations of preventive interventions to address internet addiction in young children. In addition, this study provided recommendations on future intervention study opportunities to overcome the limitations of the existing interventions.

## Methods

### Overview

This review was conducted following the PRISMA (Preferred Reporting Items for Systematic Reviews and Meta-Analyses) guidelines to identify, report, and synthesize the evidence systematically [43]. We performed the systematic literature review in six key stages as follows: (1) determining the research questions, (2) defining the search strategy and conducting the literature search, (3) selecting the relevant studies, (4) assessing the risk of bias, (5) extracting the data, and (6) analyzing and reporting the data.

### Research Questions

We formulated two research questions to achieve the objective of this study as follows: (1) What intervention approaches have been developed, and what are their efficacy outcomes to prevent or reduce internet addiction risks in children younger than 12 years? and (2) What are the strengths and limitations of the existing interventions to prevent internet addiction in children younger than 12 years?

### Search Terms and Strategy

This study reviewed primary studies discussing interventions to prevent internet addiction in young children and their efficacy outcomes. In addition, some terms are commonly used to refer to the phenomenon that could lead to internet addiction, such as problematic internet use, compulsive internet use, and excessive internet use [9,12,44-46]. In this study, we also included those related terms in the literature search to obtain comprehensive evidence on the current state of preventive interventions.

We searched for relevant literature from 4 credible research databases (Scopus, Web of Science, PubMed, and PsycINFO). The literature search was conducted within the title and abstract from the databases with the following search string: internet AND (addiction OR problematic OR compulsive OR excessive) AND (prevent* OR intervention) AND (child*). We searched for peer-reviewed journals or conference articles written in English. The search was conducted on January 5, 2024. The term “internet addiction” was initially introduced in around 1996, and the conceptualization remained relevant to date [47,48]. Therefore, we searched for relevant literature using the publication timeframe between 1996 and January 2024.

### Inclusion Criteria for Study Selection

The literature search used inclusion and exclusion criteria to select relevant studies. The inclusion criteria were as follows: (1) the intervention discussed in the study was intended to prevent internet addiction in children younger than 12 years; (2) the study discussed the intervention design and its efficacy outcomes—various types of efficacy assessments were allowed in our evidence search, such as randomized controlled trials [RCTs], quasi-experimental designs (QEDs), or other quantitative study designs; (3) the study was available in a full-text article; (4) the study was peer-reviewed; and (5) the study was written in English.

**Figure 1:**
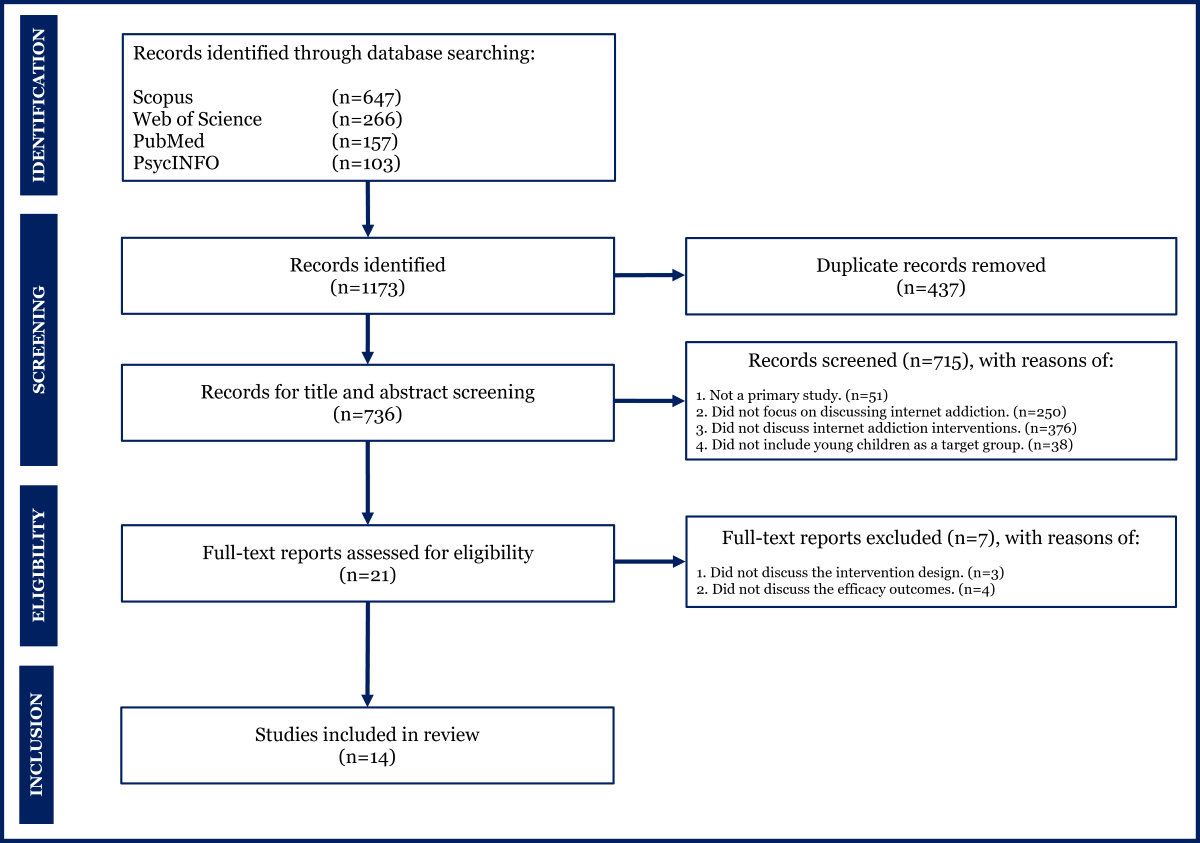
PRISMA (Preferred Reporting Items for Systematic Reviews and Meta-Analyses) flow diagram.

In this systematic review, we focused on discussing the evidence from primary studies. Therefore, we excluded some types of articles: (1) editorial, (2) review, (3) study protocol, and (4) commentary. Two authors (YT and AAM) performed the study selection, and all the authors checked the results. The screening and selection processes in this study are shown in the PRISMA flow diagram in Figure 1.

### Risk of Bias Assessment

To assess the risk of bias, we evaluated each study using the Mixed Methods Appraisal Tool (MMAT; version 2018; McGill University) [49]. This tool has been proven valid and reliable for assessing the methodological quality of empirical studies with various study designs [50]. MMAT is suitable in this study since we included primary studies with multiple study designs.

The MMAT consists of 2 screening indicators for all study types and 5 unique quality indicators for each type of study (eg, quantitative RCT, quantitative nonrandomized, quantitative descriptive, qualitative, and mixed methods) [49]. Therefore, each study design has 7 indicators to assess. However, for a mixed methods study, there are 17 indicators to assess (2 screening indicators + 5 mixed methods indicators + 5 quantitative indicators + 5 qualitative indicators). The risk-of-bias assessment was conducted on the studies that had passed the screening and selection process. The assessment was initially performed by 2 authors (YT and AAM), and the final decisions were made based on the authors’ consensus.

### Data Extraction

The data extraction aimed to summarize the included studies systematically. The PICOS (Population, Intervention, Comparison, Outcome, and Study type) framework was used to systematically report the key evidence of each study [51]. The critical information of each included study was collected, such as authors, year, country, study design (including measurement scale and timeframe), participants, intervention design, control condition, and key outcomes (Multimedia Appendix 1 [52-66]).

### Data Analysis

The included studies in this review were thematically analyzed based on their intervention characteristics, approaches, and efficacy outcomes [67]. The mechanisms, strengths, and limitations of each intervention approach were also investigated to identify gaps and directions for future preventive interventions to address the problem. Recommendations for future studies were provided based on the findings of this study. The data extraction and analysis were performed by 2 authors (YT and AAM).

## Results

### Overview of the Studies

The initial literature search found a total of 1173 articles. After screening and selecting relevant articles (Figure 1), we included 14 studies that met all the inclusion criteria. The summary of included studies based on the PICOS framework is shown in Multimedia Appendix 1.

Although we searched publications using a timeframe between 1996 and 2024, all included studies that suited our criteria were conducted after 2013. The sample size ranged from 10 to 3141. Most studies (13/14, 93%) focused on children aged between 9 and 12 years (or older) as the target group. Only 1 study was intended for children younger than 7 years. The final samples came from Europe (7/14, 50%), Asia (6/14, 42.9%), and the Middle East (1/14, 7.1%). Based on the country, most of the included studies were conducted in Turkey (n*=*5) and Hong Kong (n*=*3). The rest of the studies came from various countries, including South Korea (n*=*1), Germany (n*=*1), Lebanon (n*=*1), Norway (n*=*1), Taiwan (n*=*1), and Thailand (n*=*1).

This review explored the existing preventive interventions that have been assessed to understand the efficacy outcomes of the intervention discussed. Four testing designs were used in the studies, including RCT (n=5), QED (n=5), single-arm trial (n=3), and cross-sectional study (n=1). There was variability in the implementation duration, measurement timeframe, and scale used to measure the efficacy outcomes. The implementation of the interventions discussed in this study varied between 1 week and 6 months. The measurement timeframe was also varied between 1 week and 12 months. Some common internet addiction scales were used, such as Young’s Internet Addiction Scale (n*=*3) [8] and Korean Internet Addiction Proneness Scale (n*=*2) [68].

The interventions discussed in the included studies were aimed at preventing or reducing the risks of internet addiction (n*=*9), internet gaming addiction (n*=*6), and problematic internet use (n*=*1). Two studies claimed that their interventions were intended to prevent internet addiction and internet gaming addiction [57,58], and 1 study developed an intervention to prevent both internet addiction and smartphone addiction [59]. The included studies involved children’s environment (eg, families and schools) in their intervention design, such as teachers (n=7), parents (n=4), peers (n=2), and school nurses (n=1).

The included studies mentioned the theoretical underpinnings that underlie their intervention designs. The interventions used various well-established theories related to parenting (eg, parental mediation [52], positive parenting [55], and parenting styles [58]), psychosocial (eg, self-regulation [60], social cognitive [61], self-determination [62], ecological systems [62], family systems [59], and operant conditioning [58]), and learning (eg, participatory learning [60], media literacy [57], and gamified learning [53]).

### Risk of Bias Assessment

The risk of bias assessment was conducted on the 14 studies (Multimedia Appendix 2). All included studies passed the 2 screening indicators for clear research questions and data collection to address the questions. Five RCT studies were assessed based on 5 quality indicators in terms of randomization, baseline comparison, outcome data, outcome assessor, and intervention adherence. Four studies provided adequate explanations of all required quality indicators. One study did not clearly explain one indicator about the comparison of the baseline conditions between intervention and control groups [52].

Nine studies were assessed using 5 quality indicators for quantitative nonrandomized studies (including QED, single-arm trial, and cross-sectional study designs). This includes participants’ representativeness, measurement appropriateness, outcome data, confounders’ accountability, and intervention or exposure administration. Seven of 9 studies met all the quality indicators. Two studies did not provide adequate rationale about the representativeness of their samples to the target population [53,54]. One study did not clearly describe the potential confounders in their studies [54].

According to the risk of bias assessment results, 11 studies met all quality indicators, 2 did not meet 1 indicator, and 1 did not meet 2 indicators. We decided to include all the studies by taking into account their methodological limitations in extracting and analyzing the data.

### Preventive Intervention Approaches and Their Outcomes

#### Overview

The preventive interventions included in this study were categorized based on the similarity of the working mechanism to achieve the objective. Four different approaches were identified from the existing interventions. These were children’s education, parenting strategy, strategic physical activity, and counseling.

#### Children’s Education (n=5)

The interventions in this category aim to prevent or reduce internet addiction risks in young children by enhancing their knowledge or skills. The educational materials provided through this approach can be divided into two main goals as follows: (1) improving children’s digital literacy and encouraging healthy internet use (n*=*4) and (2) developing children’s competencies in combating addictive behavior (n*=*1). The interventions in this category were developed as school materials or curricula, thus involving teachers in delivering the materials to their students in classes.

Three educational interventions developed limited meetings (4-8 sessions, 30-90 minutes each) for children to enhance their knowledge and skills about digital literacy and healthy internet use: School-Based Media Literacy [57], School- and Family-based Intervention [60], and Healthy Internet Use [61]. These programs were delivered as offline seminars, training, or classes to educate children about internet use, risky online behavior, and how to prevent and anticipate internet addiction. Although the goals were similar, they used different theoretical underpinnings to develop the intervention, such as media literacy, social cognition, self-regulation, and participatory learning. Based on the efficacy assessment, these interventions showed promising efficacy in lowering the risks of internet addiction or internet gaming addiction [57,60,61].

One intervention (Wise IT-use) used a hybrid format (combining 3-month online training and an offline workshop) to deliver similar materials [53]. This intervention was developed based on gamification learning and flow theory. It provided multimedia learning and flexible online training to enhance children’s experience in learning the materials. This program showed promising efficacy in lowering the risks of internet gaming addiction (*χ*^2^_4_=42.89, *P*<.001; *d*=.5) [63].

The last intervention in this category (B.E.S.T. Teen) aimed to equip children with youth development competencies to combat addictive behavior, including cognitive, emotional, social, and behavioral competencies [63]. Similar to 3 other interventions in this category, it consisted of limited offline meetings (10 sessions, 30 minutes each) to deliver the materials in school settings. This intervention significantly lowered the odds of addictive behavior (*B*=–0.61, SE 0.19; odds ratio 0.55; *P*<.002).

#### Parenting Strategy (n=4)

This approach provides parental rules, skills, or guidelines to support parents in regulating children’s internet use. Two studies in this category developed learning materials to improve parenting knowledge and skills to prevent risky internet use in children [55,62]. The Positive Parenting Program (Triple P) provided a set of seminars (3 sessions, 2 hours each) to educate parental guidelines on how to cope with online behavior problems and health care services in young children [55]. Developed based on the positive parenting theory, this program showed promising efficacy in reducing children’s screen time and improving minor psychiatric disorders and family perception [55]. The Game Over Intervention was developed based on self-determination and ecological systems theories to provide parents with parental monitoring, parental care, and psychoeducation skills [62]. This intervention could reduce children’s screen time and addiction risks. However, the control group also showed a similar reduction, so there was not enough evidence that the intervention was better than the control group [62].

Two other studies in this category developed and discussed parenting strategies to reduce children’s internet use. The e-Discipline program used screen time as a discipline tool for parents to manage children’s behavior and attitude [58]. This program was based on parenting styles and operant conditioning theories. Through this intervention, parents reward and punish their children by adding or reducing their children’s screen time. Ironically, this intervention made their children more likely to exceed recommended screen time (2 hours a day) than before [58]. Therefore, this strategy was not promising in preventing risky internet use in children. The other intervention (Guardian Guidelines to Prevent Problematic Gaming) was developed based on the parental mediation theory [52]. This is a set of guidelines for parents or caregivers to manage children’s device use at home (eg, children should not use digital devices in their bedrooms, children should not use digital devices more than 5 days a week, and no screen time during meals). This intervention was not promising because it showed no significant difference between the intervention and control group [52]. In addition, many parents involved in this study could not understand how to implement the guidelines properly and were not consistent in implementing the guidelines.

#### Strategic Physical Activity (n=4)

The strategic physical activity approach encourages children to do more physical activities in order to become less attached to online activities. This approach could also provide many positive developments for children, such as self-regulation, executive functions, and social engagement [56,59,64].

Two interventions used sports activities to reduce the risks of internet addiction in young children. The first intervention (Intensive Sports Activity) was a program based on optimism theory where children were involved in multiple types of intensive sports activities for 12 consecutive weeks [54]. This study reported promising efficacy in reducing addiction risks (*t*(185)=20.091, *P*<.001), improving optimism (*t*(185)=–13.205, *P*<.001), and improving communication skills (*t*(185)=–14.903, *P*<.001) [54]. One similar program (Strategic Physical Activity) was a 12-week strategic basketball activity [56]. The intervention was developed based on the principle that increasing children’s executive functions would reduce their addictive behavior. However, although the intervention successfully improved motor competencies (*P*=.04; *r*=–0.38), there was not sufficient evidence that the intervention reduced the risks of addiction.

The other 2 programs in this category focused on preventing addiction by encouraging children’s physical activities through play and art activities with peers. Peer Relationship Enhancement and Traditional Children’s Game interventions were developed to encourage children to interact with their peers through playing and making art [59,64]. The first intervention used social systems and family systems theories to build the intervention, whereas the second used the psychosocial development theory as a framework. Promising efficacy was reported by the Peer Relationship Enhancement (*P*<.05; *d*=.4) and Traditional Children’s Game (*P*<.05; *d*=.77) interventions [59,64].

#### Counseling (n=1)

Counseling is professional assistance that gives advice or recommendations for coping with particular personal problems [69]. The intervention in this category prevents internet addiction through professional counseling sessions to help children reflect on their internet use, recognize their internet use problems, and find appropriate solutions. One study (Solution-Focused Intervention) was developed based on positive psychology theory to guide children in identifying problems, setting goals, and finding appropriate solutions relating to their internet use [65]. This short-term intervention (3-month implementation) consisted of 6 group interviews every 2 weeks. The intervention showed promising efficacy in lowering internet addiction risks (*P*<.01; *d*=.5) [65].

### Strengths and Limitations of the Existing Approaches

The most frequent intervention approach to prevent internet addiction in young children is children’s education (n=5). This approach showed great potential since all included studies in this category reported promising efficacy in preventing or reducing internet addiction risks. The main strength of this approach is that it provides children with understanding rather than forces them to engage in particular online behaviors (eg, reducing screen time and avoiding specific apps). In addition, this approach is flexible and can be attached to children’s daily activities, such as at school or a child community center. The challenges may appear in providing appropriate materials for the children, training the provider to deliver the materials in exciting ways, and increasing awareness to educate healthy internet use in children [8]. Besides, the family’s or parent’s roles in educating and modeling healthy internet use could also significantly influence how children can implement the materials [27,28,70]. Educational interventions typically need initial awareness and active commitment from children to be involved. In addition, the educational interventions included in this study are temporary programs (limited seminar, training, or workshop sessions).

Parents had a significant influence in providing internet access and controlling children’s online behavior [31,71,72]. However, some parenting strategy interventions included in this study were not efficacious [52,58,62]. Parents can contribute significantly to preventing internet addiction through education, role modeling, or positive relationships with children [8,27,28]. Some limitations of the parenting approach may support the outcomes of the existing interventions: (1) parents had limited capacity, capability, and consistency in implementing the strategy [52,62]; (2) children with better knowledge about technology might outsmart their parents so that they could violate parental rules easily [27,62]; (3) forcing children’s online behavior by implementing restrictions and limitations without giving proper understanding might not be effective and favorable [52,58,73]; and (4) harnessing screen time as a tool for rewarding or punishing children’s behavior might not work to prevent internet addiction [58,74].

The strategic physical activity approach showed promising efficacy outcomes in preventing addictive behavior in children. The strengths of this approach are its ability to enhance peer relationships and smoothly shift children’s attention to physical activities to reduce children’s engagement with online activities. In addition, the interventions in this approach could bring additional positive values for children, such as self-regulation, executive functions, and social engagement [56,59,64]. However, all interventions in this approach provided limited physical activity sessions for children; thus, we could not determine the sustainable effects of the interventions. Matching the physical activities with children’s interests would also be crucial because children may have various activity preferences (eg, some children may not like sports or arts).

The counseling approach utilized the capability of health practitioners to provide children with proper advice or recommendations to address internet addiction [8]. The intervention included in this review used a solution-focused approach in the counseling sessions to achieve the objective [65]. The use of counseling or psychotherapy approaches was common and promising in reducing the symptoms of internet addiction [37,38]. Several common approaches exist, such as cognitive behavioral therapy and family therapy [9,37]. However, there is a lack of discussion about the application and outcomes of those in preventing internet addiction in young children. The counseling approach needs the commitment of children and their parents or guardians to spend time, money, and energy attending the sessions. In addition, this intervention needs to be delivered by professional health practitioners, who may not always be available or accessible, especially in low-resource or rural areas [75]. Some parents or children may also have a negative stigma towards counseling or therapy because they may be considered different or “abnormal” [76].

## Discussion

### Principal Findings

This review study has provided an exploration of the current approaches, efficacy outcomes, and strengths and limitations of the existing interventions to prevent or reduce the risks of internet addiction in young children. Ten (71%) out of 14 preventive interventions for young children reported promising efficacy in preventing or reducing the risks of internet addiction. Those interventions showed small to medium effect sizes of their interventions [77]. However, 3 studies with promising efficacy did not provide effect size information in their articles (we have tried to follow up this information with the corresponding author via email).

According to the outcomes, interventions that enhance children’s knowledge and skills in having appropriate digital literacy and healthy online behavior were more likely to show promising efficacy than interventions that force children to reduce screen time. Interventions with this objective showed promising efficacy, regardless of the approaches used (eg, children’s education, parenting strategy, and counseling) [53,55,57,60,61,63,65]. Another study (Game Over Intervention) with a similar aim also reported a significant risk reduction [62]. However, there was insufficient evidence because a similar reduction was also found in the control groups. In contrast, interventions that forced children to restrict their screen time without proper education and communication were not efficacious [52,58]. It showed that instilling an awareness of healthy online behavior in children had a better effect than enforcing restrictions. Previous studies similarly found that shaping children’s behavior would be more effective than forcing them to do certain behaviors [27,78]. Forcing the children too much may also provide a negative experience [74].

Interventions that shift children’s focus from online activities to real-world activities also showed promise in reducing children’s engagement with the internet, thereby preventing addictive behavior [54,59,64]. Those interventions leveraged children’s social relationships with peers through various activities (eg, sports, plays, and arts) to prevent overengagement with online activities. Prior studies also highlighted the importance of improving peer relationships and encouraging more real-world activities in combating internet addiction in children [79-82]. In addition, it may be beneficial if the intervention can suit the physical activities with children’s or families’ preferences to increase its acceptability.

Prior studies highlighted the vital role of parents in preventing internet addiction in young children [27,28]. However, 3 parenting strategy interventions included in this study did not show promising efficacy [52,58,62]. We identified that the interventions might be ineffective for two main reasons: (1) inappropriate strategies and (2) the parents’ failure to implement the intervention as intended. Regardless of the outcomes, involving parents is essential in developing interventions to encourage healthy internet use in children [27,83]. To overcome the first limitation, we suggest further studies to collaborate with related experts and health practitioners to develop appropriate parental guidelines. Technology may also be used to provide tailoring or personalized strategies for parents based on their preferences or conditions [84,85]. To overcome the second limitation, we suggest further studies to provide adequate training and understanding for parents in implementing the strategies. Appropriate strategies would not be useful if parents cannot apply them well. Therefore, we should also consider the motivation, usability, and learnability factors when parents apply the strategies to their children.

According to the Interactional Theory of Childhood Problematic Media Use model, internet addiction in young children could be significantly influenced by factors related to the family and peers (eg, relationships, behavior, attitude, and media influence) [31]. The role of children’s environment may improve or exacerbate the risks of internet addiction in young children. Therefore, involving people who can influence children’s online behavior may be beneficial for the success of preventive interventions. The existing interventions in the included studies also involved children’s families or schools in delivering the intervention. For instance, parents were trained to manage children’s internet use, teachers delivered education materials and physical activity programs, peers collaborated to do physical activities together, and health practitioners provided professional counseling. However, no single intervention involved more than 1 stakeholder. Therefore, we recommend that the intervention design and implementation involve stakeholders that can significantly influence children’s behavior. They should be used to reinforce positive online behavior in children and prevent negative influence [86]. For example, parents or teachers can be role models or educators, peers or siblings can be social facilitators, and health practitioners can create educational materials to combat addictive behavior. Their combined contributions will create positive environments for children to prevent addictive behavior.

The findings of this study showed that each intervention approach has some limitations in design and implementation that need to be further improved. Although some interventions reported promising efficacy outcomes, most of them were temporary programs with limited sessions and accessibility (eg, seminar or training, professional counseling, and strategic physical activity). Sustainable interventions may be needed to improve long-term effects in young children [87,88]. In addition, interventions that need much money or expert involvement (eg, counseling) may not always be accessible in low-resource regions [89]. Therefore, we suggest developing interventions with better accessibility to reach various families with different backgrounds.

Combining multiple approaches may improve efficacy in overcoming the limitations of each approach [90]. For example, we may develop an integrated intervention that facilitates families in educating healthy internet use, determining appropriate internet use regulations, and suggesting attractive physical activities to prevent their children from over-engagement with online activities. The use of digital technology may be beneficial in achieving these goals [91]. Digital technology can increase the capability, efficiency, and accessibility of the intervention in encouraging children to have healthy online behavior [92,93].

Some digital tools, such as parental control or digital well-being software, have been developed to support managing children’s device use [88,92]. Parental control software could be beneficial in improving children’s online safety and parental mediation [74,94]. However, in this study, we did not find studies that investigated the design and efficacy outcomes of digital interventions or digital tools to prevent internet addiction in young children. Some studies developed digital tools to manage children’s internet use, but they were not developed specifically to prevent internet addiction, and their efficacy outcomes have not been tested [74,88,95]. Therefore, further studies are needed to investigate, develop, and evaluate appropriate digital tools to prevent internet addiction in young children.

The interventions discussed in this study mainly focused on educating or regulating young children as problem owners. However, there were concerns about digital product features or content that could encourage children to have more online activities [22,33,34]. Considering that excessive and uncontrolled online activities can cause internet addiction, we suggest future studies to investigate how product developers for children can contribute to preventing addictive behavior in their users. We encourage product developers, related experts, or policy makers to consider safe child-computer interaction in supporting internet addiction prevention in children. This can be manifested in various forms, such as making child-friendly design guidelines, interaction strategies, or policies.

In this study, we searched for evidence of the existing interventions for preventing internet addiction in children younger than 12 years. However, the interventions identified in this study mainly focused on children aged 9-12 years. There is a lack of intervention studies intended for children younger than 8 years. Since today’s children start using the internet from early childhood (1-5 years old) [3-5], we suggest investigating more intervention studies focusing on children younger than 8 years. It is crucial to ensure children do not have addictive behavior in early childhood since optimal cognitive development typically starts from that period [96,97].

### Limitations of the Study

This study may have some possible limitations. This systematic review focused on investigating relevant evidence about preventive interventions to address internet addiction in children. To date, the conceptualization between internet addiction and other related terminologies (eg, digital addiction and smartphone addiction) is still under debate due to some similarities in symptoms, mechanisms, and harmful effects [13,98,99]. Consequently, interventions for preventing related problems like digital or smartphone addiction may also have the prospect of preventing internet addiction. However, we did not include other related terms in this study to avoid biases. Future studies are needed to define and standardize this conceptualization issue before considering them as similar constructs.

In this study, we did not limit the regions where the studies were conducted to avoid selection biases. However, the studies included in this review mainly came from European and Asian countries. In this review, we did not get samples from some regions (eg, North America and Australia) due to our inclusion criteria to achieve the objectives of this particular study, which might be a limitation of our study. For instance, we initially found 15 studies with relevant topics (preventing internet addiction in young children) from North America and Australia. However, we excluded them because they did not report their intervention design or efficacy outcomes. Accordingly, this study might have limited generalizations that must be considered when applying the findings. This limitation also indicated the need for future studies to develop, implement, and evaluate new or existing preventive interventions in different regions to extend our knowledge on preventing the problem effectively in multiple contexts.

Although the existence of addiction to the internet and online gaming has been recognized as a diagnosable mental condition (eg, in *Diagnostic and Statistical Manual of Mental Disorders* and *International Statistical Classification of Diseases*), the diagnosis may have a cultural limitation. For example, in the United States, internet addiction was considered a comorbid condition, not a primary diagnosis [100], and approximately 86% of internet addiction cases were comorbid with other conditions [101]. Other studies similarly reported the possible comorbidity of internet addiction with other diagnoses such as attention deficit and hyperactivity disorder and depression [102,103]. We recognize that considering the comorbid conditions of the participants is essential to have a more accurate diagnosis of internet addiction. However, only 1 study [56] in our sample screened the participants’ comorbid conditions, which might be a limitation of our study. We suggest further internet addiction studies to pay attention to this comorbidity issue to have better results and validity.

The variability of the measurement scale and timeframe in the included studies raised challenges in comparing the efficacy outcomes of the interventions. Therefore, we could not perform a comparative analysis of the interventions in this study. Nevertheless, we have provided a deep exploration and discussion of the potential preventive intervention mechanisms and approaches as valuable insights to address this problem better in the future.

### Conclusions

There is a growing concern about internet addiction in young children due to the increasing number of childhood internet users and their vulnerability to this problem. This review study has investigated and discussed the current approaches, efficacy outcomes, and strengths and limitations of the existing interventions to prevent or reduce the risks of internet addiction in young children. This study identified 14 preventive interventions categorized into 4 groups based on their approaches to achieving the objective. This includes children’s education, parenting strategy, strategic physical activity, and counseling. Ten interventions showed promising efficacy outcomes in preventing or reducing internet addiction risks in young children with small-to-medium effect sizes.

Overall, preventive interventions that enhance children’s competencies in having appropriate online behavior and literacy were more likely to have better efficacy than interventions that force children to reduce screen time. Interventions that shift children’s focus from online activities to real-world activities also showed promise in reducing children’s engagement with the internet, thereby preventing addictive behavior. In this study, we have also identified the limitations of each intervention approach as valuable considerations in developing future interventions to address the problem. The current limitations include several domains, such as the temporariness of the program, accessibility, parental capability, and implementation.

The findings of this study suggest the need to develop more sustainable and accessible interventions in educating healthy internet use, determining appropriate internet use regulations for children, and suggesting attractive activities to prevent children from overengagement with online activities. Involving children’s stakeholders (eg, parents, teachers, and peers) can be beneficial in reinforcing positive online behavior in children and preventing negative influence. The use of technology-mediated interventions is recommended to improve the capability, efficiency, and accessibility of the intervention. Further studies are needed to investigate, develop, and evaluate appropriate digital tools to prevent internet addiction in young children. In developing parental control interventions, we must consider the appropriateness of the strategies with familial contexts or cultures and provide adequate training or understanding for parents to apply the strategies as intended. Future interventions may also emphasize the role of product developers, related experts, or policy makers by developing child-friendly product design guidelines to prevent developers from making products that can encourage overuse. Last, future studies may be needed to develop preventive interventions for children younger than 8 years. This was lacking in the current literature but urgently needed, given that today’s children start interacting with technology at a very young age.

## Appendix

Multimedia Appendix 1 Summary of included studies.

Multimedia Appendix 2 Risk-of-bias assessment summary.

Multimedia Appendix 3 PRISMA (Preferred Reporting Items for Systematic Reviews and Meta-Analyses) checklist.

## Abbreviations

MMAT: Mixed Methods Appraisal Tool
PICOS: Population, Intervention, Comparison, Outcome, and Study type
PRISMA: Preferred Reporting Items for Systematic Reviews and Meta-Analyses
QED: quasi-experimental design
RCT: randomized controlled trial
