# Supplementary material for: Preventive Interventions for Internet Addiction in Young Children: Systematic Review
Source: JMIR Ment Health. 2024 Aug 30;11:e56896. doi: 10.2196/56896 (PMC11399750; doi:10.2196/56896)
Supplement: Multimedia Appendix 1 [file mental_v11i1e56896_app1.docx]

Appendix 1. Summary of Included Studies

| No. | Authors, Year | Region | Participants | Intervention | Control | Outcomes | Study Design |
| --- | --- | --- | --- | --- | --- | --- | --- |
| 1 | Tseng et al., 2022 [1] | Taipei, Taiwan | 10 primary school children with criteria:  (a) had internet addiction risks (according to CIAS score)  (b) no eye problems  (c) no physical disabilities  (d) no comorbid disorders.  M_age_ = 10.45±0.68  Age range = 9–11  Boy:Girl = 9:1 | **Strategic Physical Activity**  This physical activity program is a 12-week basketball activity held twice a week for 90 minutes. Each session consists of the following: 1) 5 minutes of warmup, 2) 20 minutes of fitness skills, 3) 40 minutes of fundamental skills, 4) 20 minutes of game set activity, and 5) 5 minutes of cool down. The program was chosen based on physical activity guidelines by Janssen and LeBlanc, 2010 [2].  Theoretical framework:  Executive functions | n/a | There was no sufficient evidence of the program in reducing internet addiction and improving reaction time and accuracy. However, the program was significantly improved motor competencies (*p*=.04, *r*=-0.38). | SAT^e^  Scale:  - Chinese Internet Addiction Scale (CIAS)  - Movement Assessment Battery for Children (MABC-2)  - Cognitive Task  Measurement Timeline:  - Before intervention  - After 12-week intervention |
| 2 | Kacar and Ayaz-Alkaya, 2022 [3] | Turkey | 42 children from two primary and middle schools without any illness that may prevent them from playing games.  IG^a^ = 20  Age range= 8–11  5^th^:6^th^ Grade= 9:11  Boy:Girl = 6:14  CG^b^ = 22  Age range = 8–11  5^th^:6^th^ Grade = 5:17  Boy:Girl = 11:11 | **Traditional Children's Game**  This program is a school-assisted activity to help children spend their free time with appropriate activities, develop friendships, make conscious use of the internet, develop social skills, and reduce stress. The games are played in groups of ten at lunchtime and after school. The program was implemented for eight weeks, three days a week for 60 minutes.  Theoretical framework:  Psychosocial development | Participants in the control group played their usual games in their daily routines with no intervention. | There was a significant reduction in internet use and internet addiction risks after the intervention. There was a significant increase in social skills after the intervention. There was a significant difference in internet use, internet addiction risks, and social skills between participants in the intervention and control groups after the intervention.  (*p*<.05, *d*=.77) | QED^d^  Scale:  - Parent-Child Internet Addiction Test (PCIAT)  - Social Skills Assessment Scale  - Perceived Stress Scale  Measurement Timeline:  - Before intervention  - After 8-week intervention |
| 3 | Jo and Bang, 2022 [4] | South Korea | 52 children from eight community children's centers in city S  IG^a^ = 27  Age range = 9–12  5^th^:6^th^ Grade = 14:13  Boy:Girl = 8:19  CG^b^ = 25  Age range = 9–12  5^th^:6^th^ Grade = 10:15  Boy:Girl = 11:14 | **Peer Relationship Enhancement**  This program is a set of social activities that aim to enhance peer relationships, improve self-control, and reduce depression in children. The program consisted of group-based play and art activities during 12 sessions (1 session per week). Each session was 90 minutes long.  Theoretical framework:  Social systems and family systems | Participants in the control group received no intervention. | The intervention group showed a significant effect in reducing addiction risks, reducing depression, and increasing peer relationships (*p*<.05, *d*=.4). However, no effect was found on self-control. | QED^d^  Scale:  - Integration of the internet (K-scale) and smartphone (S-scale) addiction scales.  - Peer Relational Skills Scale (PRSS)  - Depression Scale for Children (CES-DC 11)  - Self-Control Rating Scale (SCRS)  Measurement Timeline:  - Before intervention  - After 12-week intervention  - Two months after intervention |
| 4 | Akgül-Gündoğdu & Selçuk-Tosun, 2022 [5] | Sivas, Turkey | 128 children with internet addiction risks (YIAS score >30)  IG^a^ = 64  M_age_ = 11.34±1.12  Age range = 10–15  Boy:Girl = 42:22  CG^b^ = 64  M_age_ = 11.98±1.18  Age range = 10–15  Boy:Girl = 39:25 | **Solution-Focused Intervention**  This program involves short-term counseling through group meetings to help children identify problems and solutions relating to their internet use. The stages of SFA are problem recognizing and understanding, goal setting, solution questioning, and solution rating and reframing. The counseling session consists of six group interviews every two weeks.  Theoretical framework:  Positive psychology | Participants in the control group received no intervention. | The study showed that the internet addiction score of the intervention group was significantly lower after the intervention (*p*<.01, *d*=.5). In addition, the nutrition–exercise attitude and behavior scores were significantly increased in the post-test (*p*<.05). | RCT^c^  Scale:  - Young Internet Addiction Scale (YIAS)  - Nutrition–Exercise Behavior Scale  - Nutrition–Exercise Attitude Scale  Measurement Timeline:  - Before intervention  - After 3-month intervention |
| 5 | Sari & Karagün, 2019 [6] | Kocaeli, Turkey | 186 children from sports schools with internet addiction risks (showed symptoms of internet addiction)  M_age_ = 10.82±0.88  Age range = 10–12  4^th^:5^th^:6^th^ Grade = 92:36:58  Boy:Girl = 78:108 | **Intensive Sports Activity**  This physical activity program is a set of intensive sports activities to help children detach from their internet dependency. During the 12 weeks, children should be involved in many sports activities.  Theoretical framework:  Optimism | n/a | The program significantly reduced non-chronic internet addiction risks (*t_(185)_*=20.091, *p*<.001). In addition, it showed a significant increase in optimism (*t_(185)_*=-13.205, *p*<.001) and communication skills (*t_(185)_*=-14.903, *p*<.001). | SAT^e^  Scale:  - Young Internet Addiction Scale (YIAS)  - Optimism Scale  - Communication Skills Scale  Measurement Timeline:  - Before intervention  - After 12-week intervention |
| 6 | Li et al., 2019 [7] | Hong Kong | 362 parents of children with regular online gaming activities from 39 primary schools  IG^a^ = 163  M_age_ = 10.22±1.01  Age range = 8–12  Boy:Girl = 103:60  Parent’s M_age_ = 42.75±5.94  Father:Mother = 23:140  CG^b^ = 199  M_age_ = 9.97±0.95  Age range = 8–12  Boy:Girl = 121:78  Parent’s M_age_ = 41.89±5.71  Father:Mother = 12:187 | **Game Over Intervention (GOI)**  This program is parent-based learning materials to provide parents with the knowledge, skills, and attitudes to enhance positive parenting and family environments. The program consists of three modules: parental monitoring, parental care, and psychoeducation.  Theoretical framework:  Self-determination and ecological systems | Participants in the control group did not receive the intervention materials. During the implementation period, they received different educational materials (effective learning in children). | There was a significant reduction in children's gaming time (*p*<.001), exposure to violent games (*p*<.001), and symptoms of internet gaming disorder (*p*<.001). However, there was no significant difference between the intervention and control group for exposure to violent games (*p*=.13) and symptoms of internet gaming disorder (*p*=.17). Therefore, there was no sufficient evidence that the program was better than the control group in lowering addiction. | RCT^c^  Scale:  - Korean Internet Addiction Proneness Scale (KS-II)  - Average gaming time  - Exposure to violent games  Measurement Timeline:  - Before intervention  - One week after intervention  - Three months after intervention |
| 7 | Chau et al., 2019 [8] | Hong Kong | 248 children from four primary schools  M_age_ = 10.16±0.97  Age range = 7–13  Boy:Girl = 139:109 | **Wise IT-use (WIT)**  This educational program is a 3-month prevention program in the form of online multimedia training and an offline workshop to enhance children's awareness and knowledge of internet gaming disorder and risky online behaviors. The program consists of three main parts: 1) introduction to the topics, 2) case studies about the unfavorable consequences, and 3) effective ways to address the problem.  Theoretical frameworks:  Gamification learning and flow theory | n/a | The symptoms of Internet Gaming Addiction and the proportion of students at risk of Internet Gaming Addiction were significantly reduced after the program ($X^{2}$=42.89, *p*<.001, *d*=.5). In addition, the intervention showed a significant reduction in negative affect and social anxiety (*p*<.001) and increase in positive affect (*p*=.03). However, there was no significant reduction in risky online behavior (*p*=.09). | SAT^e^  Scale:  - Korean Internet Addiction Proneness Scale  - Risky Online Behavior Inventory  - Positive and Negative Affect Schedule (PANAS)  - Social Anxiety Scale  - Loneliness Scale  Measurement Timeline:  - One month before intervention  - Two months after intervention |
| 8 | Özyurt et al., 2018 [9] | Cappadocia , Turkey | 76 mothers of children from hospitals and public health centers  Child’s M_age_ = 4.10±1.03  Age range = 3–6  Boy:Girl = 40:36  Maternal age:  20–30 years old = 48  >30 years old = 28  Mother’s working status:  Housewife = 40  Working = 36 | **Positive Parenting Program (Triple P)**  The Triple P is a set of seminars that guides essential healthcare services and moderate behavioral problems. The seminars include primary interventions to provide behavioral guidance for children with mild behavioral problems with parents. The study conducted the program in three sessions (1 session per week, two hours per session).  Theoretical framework:  Positive parenting | n/a | There was a significant decrease in the percentage of children with excessive digital device duration after the intervention. In addition, there was a significant improvement in GHQ (*p*=.001) and FAD subscales: behavior control (*p*=.002), affective involvement (*p*=.01), affective responsiveness (*p*=.037), and roles (*p*=.022).  Comparison of device use:  TV > 5 hours per week: 39.6% (*n*=30) to 3.9% (*n*=3)  Computer > 3 hours per week: 31.58% (*n*=24) to 10.4% (*n*=8)  Smartphone > 2 hours per week: 30.3% (*n*=23) to 9.2% (*n*=7) | QED^d^  Scale:  - Digital Device Use Duration  - General Health Questionnaire (GHQ)  - The McMaster family assessment device (FAD)  Measurement Timeline:  - Before intervention  - After 3-week intervention |
| 9 | Uysal & Balci, 2018 [10] | Turkey | 84 children from two primary schools with internet addiction risks (YIAS score >90)  IG^a^ = 41  M_age_ = 10.98±0.68  Age range = 11–16  Boy:Girl = 31:10  6^th^:7^th^:8^th^ Grade = 14:14:13  CG^b^ = 43  M_age_= 11.43±0.97  Age range = 11–16  Boy:Girl = 28:15  6^th^:7^th^:8^th^ Grade = 12:16:15 | **Healthy Internet Use Program**  This 3-week training program consists of eight sessions. The material given includes healthy Internet use, self-expression, self-recognition, the effects of internet addiction, a sedentary lifestyle on Internet use, internet addiction problems, overcoming internet addiction, and building awareness of behavior changes.  Theoretical framework:  Social cognitive | Participants in the control group received no intervention. | The evaluation showed a significant addiction score decrease in the intervention group after the intervention ($X^{2}$=84.089, *p*<.001). The control group also showed a significant decrease ($X^{2}$=55.632, *p*<.001). There was also a significant difference in addiction scores between the intervention and control groups after three months (*p*=.014) and nine months (*p*<.001). | RCT^c^  Scale:  Young Internet Addiction Scale (YIAS)  Measurement Timeline:  - Before intervention  - Three months after intervention  - Nine months after intervention |
| 10 | Krossbakken et al., 2018 [11] | Norway | 1,657 parents of children from the Norwegian Population Registry  M_age_ = 10.1  Age range = 8–12  Boy:Girl:n/a = 876:759:22  Father:Mother:Other Family Members = 583:1,022:52  IG^a^ = 831  CG^b^ = 826 | **Guardian Guidelines to Prevent Problematic Gaming**  This parenting strategy is a set of guidelines for the child's guardian to manage their digital device use at home, such as screen time management, device position, parental rules, valuable information, parent-child communication, and more.  Theoretical framework:  Parental mediation | Participants in the control group received no intervention. | There was no significant difference between the intervention and control group in internet gaming disorder risks, sleep habits, parental mediation, and parental efficacy.  (*α*=.05, *d*=.2) | RCT^c^  Scale:  - Internet Gaming Disorder Scale  - Child Sleep Habits Questionnaire (CSHQ)  - Parental Mediation Scale  - Parental Efficacy Scale  Measurement Timeline:  - After 4-month intervention  - After 6-month intervention |
| 11 | Apisitwasana et al., 2018 [12] | Bangkok, Thailand | 310 primary school children  IG^a^ = 151  M_age_ = 9.77±0.79  Age range = 9–11  Boy:Girl = 73:78  4^th^:5^th^ Grade = 81:70  CG^b^ = 159  M_age_ = 10.05±0.67  Age range = 9–11  Boy:Girl = 93:66  4^th^:5^th^ Grade = 81:78 | **School-&Family-Based Intervention**  This educational program is a set of learning materials to address children’s knowledge and self-regulation skills to manage the frequency and time spent on internet gaming activity. The program contained 1-hour classes during eight weeks (one class per week).  Theoretical frameworks:  Self-regulation and participatory learning | Participants in the control group received no intervention. | There were significant differences in knowledge, self-regulation, and internet gaming addiction behaviors immediately and after three months.  (*p*<.05, f=.1) | QED^d^  Scale:  - Game Addiction Screening Test (GAST)  - Game Addiction Protection Scale (GAPS)  Measurement Timeline:  - Before intervention  - After 8-week intervention  - Three months after intervention |
| 12 | Shek et al., 2016 [13] | Hong Kong | 1,246 children from 5 primary schools  IG^a^ = 679  M_age_ = 11.75±0.86  Age range = 10–12  Boy:Girl:n/a = 219:214:246  5^th^:6^th^ Grade = 382:297  CG^b^ = 567  M_age_ = 11.99±0.81  Age range = 10–12  Boy:Girl:n/a = 218:191:158  5^th^:6^th^ Grade = 234:333 | **B.E.S.T. Teen**  The B.E.S.T. Teen program is an educational curriculum that promotes five components related to positive youth development: 1) nature of addiction, 2) cognitive competence, 3) emotional competence, 4) promote social competence, and 5) behavioral competence. Each component is divided into two units, each taking 30 min. The total duration of this program is 5 hours for a total of ten units.  Theoretical framework:  Positive youth development | Participants in the control group did not participate in the program. | The intervention group showed a lower occurrence of the addictive use of the internet (*B*=−0.61, *S.E.*=0.19, *OR*=0.55, *p*<.002). In addition, the intervention showed significant prediction on the intention of internet overuse in the coming two years (*β*=−.07, *p*=.02). However, no significant effect was identified from other indicators. Therefore, the evaluation showed partial evidence of the program's effectiveness. | QED^d^  Scale:  A self-developed scale (57 items) measuring addictive behavior, behavioral intention, psychosocial competencies, and knowledge and beliefs about addiction.  Measurement Timeline:  - Before intervention  - After intervention |
| 13 | Hawi & Rupert, 2015 [14] | Lebanon | 3,141 children from 15 private primary schools  M_age_ = 9.02±1.26  Age range = 7–11  Boy:Girl = 1,588:1,553 | **e-Discipline**  This parenting strategy uses screen time as a discipline tool to educate children. Through this intervention, children's desirable behaviors will be rewarded with more screen, whereas their undesirable behaviors will be punished with screen time reduction.  Theoretical frameworks:  Parenting styles and operant conditioning | n/a | Children whose parents implemented the intervention were more likely to exceed the recommended screen time (2 hours per day). Odds Ratios and Percentages of Children:  Boys: Reward and Punishment (13.4%, *OR*=2.244, *p*<.001); Reward (21.0%, *OR*=1.672, *p*<.001); Punishment (41.6%, *OR*=1.435, *p*<.01)  Girls: Reward and Punishment (8.4%, *OR*=1.551, *p*<.05); Reward (16.3%, *OR*=1.314, *p*<.05); Punishment (33.1%, *OR*=1.646, *p*<.001) | CSS^f^  Scale:  - The odds of exceeding the recommended screen time (2 hours per day)  - Screen time behavior  Measurement Timeline:  n/a |
| 14 | Walther et al., 2014 [15] | Germany | 1,843 children from 27 schools  IG^a^ = 804  M_age_ = 11.80±0.80  Age range = 10–14  Boy:Girl = 381:423  CG^b^ = 1,039  M_age_ = 12.00±0.83  Age range = 10–14  Boy:Girl = 531:508 | **School-Based Media Literacy: Vernetzte www.Welten**  This educational program is a curriculum package on self-monitoring, discussion, and reflection on children’s media use. It consists of four units (90 minutes for each unit): 1) Internet use, 2) online communication, 3) gaming behaviors and preferences, and 4) gambling. Teachers from participating schools delivered the materials during class time.  Theoretical framework:  Media literacy | Participants in the control group attended regular classes with no intervention. | There was a significant effect in lowering the gaming frequency (*β*=−1.10 [95% CI −2.06, −0.13]), gaming time (*β*=−0.27 [95% CI −0.40, −0.14]), proportions of excessive gamers (*AOR*=0.21 [95% CI 0.08, 0.57]), and the Internet Addiction Scale (*β*=−0.06 [95% CI −0.10, −0.01]). However, no effect was found on internet time spent and parental media behavior. | RCT^c^  Scale:  - German Computer Gaming Addiction Scale (KFN-CSAS-II)  - German Internet Addiction Scale (ISS)  Measurement Timeline:  - Before intervention  - After 3-month intervention  - 12 months after intervention |
| ^a^IG: Intervention Group | | | | | | | |
| ^b^CG: Control Group | | | | | | | |
| ^c^RCT: Randomized Controlled Trial | | | | | | | |
| ^d^QED: Quasi-Experimental Design | | | | | | | |
| ^e^SAT: Single-Arm Trial | | | | | | | |
| ^f^CSS: Cross-Sectional Study | | | | | | | |

References:

1. Tseng Y, Chao HH, Hung CL. Effect of a strategic physical activity program on cognitive flexibility among children with internet addiction: a pilot study. Children (Basel). 2022;9(6):798. [FREE Full text] [doi: 10.3390/children9060798] [Medline: 35740735]
2. Janssen I, Leblanc AG. Systematic review of the health benefits of physical activity and fitness in school-aged children and youth. Int J Behav Nutr Phys Act. 2010;7:40. [FREE Full text] [doi: 10.1186/1479-5868-7-40] [Medline: 20459784]
3. Kacar D, Ayaz-Alkaya S. The effect of traditional children's games on internet addiction, social skills and stress level. Arch Psychiatr Nurs. 2022;40:50-55. [doi: 10.1016/j.apnu.2022.04.007] [Medline: 36064245]
4. Jo J, Bang KS. The effect of peer relationship enhancement programs on the prevention of smartphone addiction among late school-age children in South Korea. J Pediatr Nurs. 2022;63:e127-e135. [doi: 10.1016/j.pedn.2021.09.025] [Medline: 34625302]
5. Akgül-Gündoğdu N, Selçuk-Tosun A. Effect of solution-focused approach on problematic internet use, health behaviors in schoolchildren. J Pediatr Nurs. 2023;68:e43-e49. [doi: 10.1016/j.pedn.2022.11.002] [Medline: 36446694]
6. Sari S, Karagun E. The effect of sports on children's internet addiction, optimism and communication skills. Int J Appl Exerc Physiol. 2020;9(11):157-166. [FREE Full text]
7. Li AYL, Chau CL, Cheng C. Development and validation of a parent-based program for preventing gaming disorder: the game over intervention. Int J Environ Res Public Health. 2019;16(11):1984. [FREE Full text] [doi: 10.3390/ijerph16111984] [Medline: 31167457]
8. Chau CL, Tsui YYY, Cheng C. Gamification for internet gaming disorder prevention: evaluation of a wise IT-Use (WIT) program for Hong Kong primary students. Front Psychol. 2019;10:2468. [FREE Full text] [doi: 10.3389/fpsyg.2019.02468] [Medline: 31736842]
9. Özyurt G, Dinsever C, Çalişkan Z, Evgin D. Effects of triple p on digital technological device use in preschool children. J Child Fam Stud. 2017;27(1):280-289. [doi: 10.1007/s10826-017-0882-6]
10. Uysal G, Balci S. Evaluation of a school-based program for internet addiction of adolescents in Turkey. J Addict Nurs. 2018;29(1):43-49. [doi: 10.1097/JAN.0000000000000211] [Medline: 29505460]
11. Krossbakken E, Torsheim T, Mentzoni RA, King DL, Bjorvatn B, Lorvik IM, et al. The effectiveness of a parental guide for prevention of problematic video gaming in children: a public health randomized controlled intervention study. J Behav Addict. 2018;7(1):52-61. [FREE Full text] [doi: 10.1556/2006.6.2017.087] [Medline: 29313731]
12. Apisitwasana N, Perngparn U, Cottler LB. Effectiveness of school- and family-based interventions to prevent gaming addiction among grades 4-5 students in Bangkok, Thailand. Psychol Res Behav Manag. 2018;11:103-115. [FREE Full text] [doi: 10.2147/PRBM.S145868] [Medline: 29695939]
13. Shek DTL, Yu L, Leung H, Wu FKY, Law MYM. Development, implementation, and evaluation of a multi-addiction prevention program for primary school students in Hong Kong: the B.E.S.T. teen program. Asian J Gambl Issues Public Health. 2016;6(1):5. [FREE Full text] [doi: 10.1186/s40405-016-0014-z] [Medline: 27630812]
14. Hawi NS, Rupert MS. Impact of e-Discipline on children's screen time. Cyberpsychol Behav Soc Netw. 2015;18(6):337-342. [FREE Full text] [doi: 10.1089/cyber.2014.0608] [Medline: 26075921]
15. Walther B, Hanewinkel R, Morgenstern M. Effects of a brief school-based media literacy intervention on digital media use in adolescents: cluster randomized controlled trial. Cyberpsychol Behav Soc Netw. 2014;17(9):616-623. [doi: 10.1089/cyber.2014.0173] [Medline: 25126888]
