# Supplementary material for: Preventive Interventions for Internet Addiction in Young Children: Systematic Review
Source: JMIR Ment Health. 2024 Aug 30;11:e56896. doi: 10.2196/56896 (PMC11399750; doi:10.2196/56896)
Supplement: Multimedia Appendix 2 [file mental_v11i1e56896_app2.docx]

Appendix 2. Risk of Bias Assessment Summary

| **Indicator \ Study** | **No.** | **1** | **2** | **3** | **4** | **5** | **6** | **7** | **8** | **9** | **10** | **11** | **12** | **13** | **14** |
| --- | --- | --- | --- | --- | --- | --- | --- | --- | --- | --- | --- | --- | --- | --- | --- |
|  | **Study Design** | **SAT**^c^ | **QED**^b^ | **QED**^b^ | **RCT**^a^ | **SAT**^c^ | **RCT**^a^ | **SAT**^c^ | **QED**^b^ | **RCT**^a^ | **RCT**^a^ | **QED**^b^ | **QED**^b^ | **CSS**^d^ | **RCT**^a^ |
| **Screening Questions** | |  |  |  |  |  |  |  |  |  |  |  |  |  |  |
| S1. Are there clear research questions? | | Yes | Yes | Yes | Yes | Yes | Yes | Yes | Yes | Yes | Yes | Yes | Yes | Yes | Yes |
| S2. Do the collected data allow the researcher to address the research questions? | | Yes | Yes | Yes | Yes | Yes | Yes | Yes | Yes | Yes | Yes | Yes | Yes | Yes | Yes |
|  | |  |  |  |  |  |  |  |  |  |  |  |  |  |  |
| **Quantitative randomized controlled trials** | |  |  |  |  |  |  |  |  |  |  |  |  |  |  |
| 1. Is randomization appropriately performed? | | - | - | - | Yes | - | Yes | - | - | Yes | Yes | - | - | - | Yes |
| 2. Are the groups comparable at baseline? | | - | - | - | Yes | - | Yes | - | - | No | Yes | - | - | - | Yes |
| 3. Are there complete outcome data? | | - | - | - | Yes | - | Yes | - | - | Yes | Yes | - | - | - | Yes |
| 4. Are outcome assessors blinded to the  intervention provided? | | - | - | - | Yes | - | Yes | - | - | Yes | Yes | - | - | - | Yes |
| 5 Did the participants adhere to the assigned intervention? | | - | - | - | Yes | - | Yes | - | - | Yes | Yes | - | - | - | Yes |
|  | |  |  |  |  |  |  |  |  |  |  |  |  |  |  |
| **Quantitative non-randomized** | |  |  |  |  |  |  |  |  |  |  |  |  |  |  |
| 1. Are the participants representative of the target population? | | Yes | Yes | Yes | - | Cannot Tell | - | Cannot Tell | Yes | - | - | Yes | Yes | Yes | - |
| 2. Are measurements appropriate regarding the outcome and intervention (or exposure)? | | Yes | Yes | Yes | - | Yes | - | Yes | Yes | - | - | Yes | Yes | Yes | - |
| 3. Are there complete outcome data? | | Yes | Yes | Yes | - | Yes | - | Yes | Yes | - | - | Yes | Yes | Yes | - |
| 4. Are the confounders accounted for in the design and analysis? | | Yes | Yes | Yes | - | No | - | Yes | Yes | - | - | Yes | Yes | Yes | - |
| 5. During the study period, is the intervention administered (or exposure occurred) as intended? | | Yes | Yes | Yes | - | Yes | - | Yes | Yes | - | - | Yes | Yes | Yes | - |
| ^a^RCT: Randomized Controlled Trial | |  |  |  |  |  |  |  |  |  |  |  |  |  |  |
| ^b^QED: Quasi-Experimental Design | |  |  |  |  |  |  |  |  |  |  |  |  |  |  |
| ^c^SAT: Single-Arm Trial | |  |  |  |  |  |  |  |  |  |  |  |  |  |  |
| ^d^CSS: Cross-Sectional Study | |  |  |  |  |  |  |  |  |  |  |  |  |  |  |
